# The health effects of radon and uranium on the population of Kazakhstan

**DOI:** 10.1186/s41021-015-0019-3

**Published:** 2015-10-01

**Authors:** Rakmetkazhy I. Bersimbaev, Olga Bulgakova

**Affiliations:** Institute of Cell Biology and Biotechnology, Department of General Biology and Genomics, L.N.Gumilyov Eurasian National University, Munaitpassov str.5, 010008 Astana, Kazakhstan

**Keywords:** Lung cancer, Cancer risk, Radon, Uranium, Radioactivity screening

## Abstract

The radioactive contamination is a significant factor affecting the environment and human health. Radon and its decay products are the major contributors to human exposure from natural radiation sources. World Health Organization has identified the chronic residential exposure to radon and its decay products as the second cause of lung cancer after tobacco consumption and also as the main risk-factor in never smokers. The high levels of radon are observed in the North and East areas of Kazakhstan because of the natural radiation sources and the long-term and large-scale mining of uranium. The genotoxic effects of radon on population of Kazakhstan are poorly understood, in spite of the fact that many regions of the country contain the high levels of radon. Studies elucidating potential health risk among population exposed to radon and genotoxic effect of radon in Kazakhstan are very limited or they have never been addressed in some areas. In this review, we are presenting available data on the residential radon exposure of humans in uranium mining and milling areas in the North and East areas of Kazakhstan.

## Introduction

Kazakhstan is the second largest republic by the territory among the former New Independent State countries. It has rich natural resources such as minerals, metal ores, natural gas and oil reserves. Such vast resources have stimulated the former Soviet Union to develop specific and intensive industries in the territory. Now it is well-known that the Semipalatinsk region in the north-eastern Kazakhstan was used for testing nuclear weapons for the Soviet army during the period 1949–1989 [1–3]. During the testing period, several hundred thousand people in Semipalatinsk, the Eastern Kazakhstan, Pavlodar regions of Kazakhstan and Altay region of Russian Federation were repeatedly exposed to radioactive fallout [4]. Radionuclides emanating from these tests resulted in atmospheric and environmental contaminations leading to various levels of acute and chronic radiation exposures. Long-lived radionuclides such as strontium and plutonium are present in these areas. Health effects of radiation released as a result of the tests remain as a serious concern [4, 5].

Uranium also has been actively mined and milled in the Republic of Kazakhstan [6]. Uranium ore mining and processing were initiated in Kazakhstan shortly after Second World War, and lasted for almost half a century. It has been estimated that during the Soviet period about 30–40 % of the uranium was extracted from the Asian region [7, 8].

Kazakhstan has 12 % of the world’s uranium resources and an expanding mining sector, producing about 22,830 tonnes in 2014, and planning for further increase to 2018 [9]. The mining and milling of uranium ore in the North and East Kazakhstan have caused contamination of the environment through a number of activities, specifically the open pit mining process, transportation to and from milling sites, the milling and processing of ore, and open-air storage of radioactive and non-radioactive mining wastes.

Kazakhstan might be exposed to a variety of hazardous materials including radon, a radioactive gas occurring naturally as an indirect decay product of uranium. The main concern in Kazakhstan is that approximately 85 % of the urban population lives in territories where environmental pollution is known to be exceeded permissible standards [10, 11] together with vastly unknown contamination problems. The role of radon and its radioactive decay products as human carcinogens has been established by the International Agency for Research on Cancer in 1988 and is supported by the experimental evidences obtained by the laboratory experiments on animals [12] and epidemiologic studies in humans [13]. In 2009, the World Health Organization identified the chronic residential exposure to radon and its decay products as the second cause of lung cancer after tobacco consumption and as the main risk-factor in never smokers [14]. The problem of the health-risk exposed to radon is not sufficiently studied on the populations of Kazakhstan, although the country has the regions containing the high levels of radon [15]. From this short information, it can be concluded that one of the priorities of biomedical sciences of Kazakhstan is the investigation of environmental health concerns that can be caused by known and potentially unknown environmental hazardous substances. Taking into account the importance of residential radon as a risk factor for lung cancer, in this review we are presenting available data on radon/uranium exposure of human in uranium mining and milling areas in North and East of Kazakhstan.

### Uranium sources in Kazakhstan

As noted before the Republic of Kazakhstan is a leader in the world reserves of uranium ores. Kazakhstan has 12 % of the world’s uranium resources and an expanding mining sector, producing about 22,830 tons in 2014 [9]. In 2009 it became the world’s leading uranium producer, with almost 28 % of world production, then 38 % in 2013 [9]. The largest regions enriched with the uranium deposits are located within the North Kazakhstan containing approximately 16.4 % of the uranium resources of Republic [16]. Besides, there are also large storage facilities for the radioactive waste in the North and West Kazakhstan. The total area exposed to radioactive waste from the uranium industry enterprises is estimated to approximately 100,000 hectares with a total activity of 250,000 Curie. In mining, the uranium and its decay products buried deep in the earth are brought to the surface, and the rock containing them is crushed into fine sand. After the uranium is chemically removed, the sand is stored in huge reservoirs [17]. Conservation and liquidation of uranium deposits in northern Kazakhstan was completed in 2008 in accordance with the State program “Preservation of non-uranium mining enterprises and liquidation of the consequences of uranium deposits for 2001-2010” [18].

Natural background radiation in regions of Kazakhstan is highly diverse and on average it is 3.1 mSv/year [19]. The population additionally receives about 1.1 mSv/year during the medical procedures. Thus, the total dose of artificial and natural radiation in the average per person in Kazakhstan is about 4 mSv/year, which is one and a half times higher than the world’s average level [20]. The annual effective dose of the population living near radioactively contaminated territories in North Kazakhstan was very high. For example, these doses were about 7.9 and 8.1 mSv/year in two settlements “Aksu” and “Saumakol”, respectively [10].

Uranium (U) is a radioactive chemical element of group III of the periodic system with atomic number 92 that belongs to the family of actinides. Uranium is readily soluble in water, easily reacts with the basic elements of organic compounds. In nature, uranium is found as uranium-238, uranium-235, and a very small amount of uranium-234. Uranium is not considered carcinogenic in humans, and direct evidence on the carcinogenicity in humans from different types of uranium, compounds and isotopes is limited [21]. However, depleted uranium has a chemical toxicity that is independent of its radioactivity [22]. The uranium affects all organs and tissues, as a cell poison. Large doses of uranium cause damage to the kidneys [23]. Uranium may also be associated with genotoxicity effects. At a molecular level, uranium and other metals may also induce genomic instability by affecting pathways like DNA repair, regulation of nuclear transcription factors, gene expression regulation, apoptosis, cell growth, reactive oxygen species (ROS) generation by replacing essential metals in their metabolic pathways [24]. All these effects may lead to the development of serious diseases such as cancer. Epidemiological studies of uranium miners showed a strong association of radon exposure and lung cancer risk [25].

### Health risks of radon

Radon is a chemically inert radioactive gas, occurring naturally as an indirect decay product of uranium. The most stable isotope is ^222^Rn, which is a decay product of ^238^U and ^220^Rn occurs in the decay chain for ^232^Th. Contribution to the total radiation dose from ^222^Rn is approximately 20 times greater than those of ^220^Rn, but for convenience and on the advice of United Nations Scientific Committee on the Effects of Atomic Radiation (UNSCEAR) isotopes are not differentiated and are considered together. This implies that most of the radiation is not so much from the radon as from its decay daughter products [26]. It was established that alpha-particle emissions from inhaled radon progeny cause lung cancer [27]. According to World Health Organization [14] epidemiological studies have provided convincing evidence of an association between indoor radon exposure and lung cancer, even at the relatively low radon levels commonly found in residential buildings. Radiation dose due to radon and progeny depends on concentration, particle size distribution, respiratory deposition, and lung clearance [25].

Radon comes into the residential rooms through the foundation or standing out from the building materials. As a result, there can be high levels of radiation in the room. Furthermore, radon enters into the house via tap water and cooking gas. The contribution of radon in the formation of an average human dose is estimated to be more than 50 %, so the radionuclides of radon are responsible for more than a half of the total dose of radiation, which receives an average human body from natural and man-made radionuclides environment [25].

The general effects of radon to the human body are caused by its radioactivity and it consequently carries a risk of radiation-induced cancer. In the studies of uranium miners, the workers who were exposed to the high levels of radon have shown an increased frequency of chromosomal aberrations in blood lymphocytes. Many studies have shown a correlation between the frequency of chromosomal abnormalities and a cancer risk [28–30]. Popp et al. [28] studied the chromosomal aberrations as biomarkers of genetic damage in blood lymphocytes of the former East German uranium miners who have been exposed to radon for estimation health risk for lung carcinogenesis. The authors found an increase of the frequency of chromosomal aberrations in blood lymphocytes of radon exposed population in comparison with control population. In experiments of Wolf et al. [29], it was demonstrated an increase in chromosomal aberrations in human peripheral lymphocytes after *in vitro* exposure to 18 cGy of radon and its progeny.

Increased risk of lung cancer in radon-exposed miners with elevated frequency of chromosomal aberrations was demonstrated by Smerhovsky et al. [30]. By using the Cox regression models, which accounted for the age at time of first cytogenetic assay, radon exposure they showed strong and statistically significant associations between cancer incidence and frequency of aberrant cells, respectively. A 1 % increase in the frequency of aberrant cells was paralleled by 62 % increase of cancer. A causal relationship between radon exposure and lung cancer is defined also by many other authors [31–33]. This is confirmed by numerous studies of lung cancer mortality in uranium miners groups due to radon [34, 35].

In the literature there are different data on the histological types of lung cancer induced by radon. Some studies have reported that radon increases the risk of small-cell lung cancer. Barros-Dios and colleagues [36] found that less frequent histologic types (including large cell carcinomas), followed by small cell lung cancer, had the highest risk associated with radon exposure. According to the study of Taeger et al. [37] a small cell lung cancer and squamous carcinoma is associated with the radon exposure, but adenocarcinoma is associated less. Some studies have found a positive correlation between the incidence of adenocarcinoma in the group of non-smoking women and the increasing concentration of radon in the living room [38].

Some studies have showed a statistically significant relation between cumulative radon exposure and the risk of extra pulmonary cancers [39, 40]. However, most of researchers think that the risk factor for bronchial tubes by radon inhalation is much higher than for the other organs of the human body. The main targets for the cancer induction are the segmental bronchia [41].

Radon exposure is considered the first cause of lung cancer in never smokers [42]. The aim of the study of Torres-Duran et al. [42] was to assess the effect of residential radon exposure on the risk of lung cancer in never-smokers and to ascertain if environmental tobacco smoke modifies the effect of residential radon. The authors demonstrated that individuals exposed to environmental tobacco smoke and to radon concentrations >200 Bq.m^3^ had higher lung cancer risk than those exposed to lower radon concentrations and exposed to environmental tobacco smoke. Kreuzer et al. [43] in their studies also showed that lung-cancer risk associated with radon exposure in never smokers.

Radon can enhance effects of other factors such as cigarette smoke, dust, and exhaust gases. Tobacco smoke increases the oncogenic effect of radon by 2 to 10 times and, most importantly, radon significantly reduces the latency period for lung cancer [44, 45]. Studies conducted by the United States Environmental Protection Agency showed that radon-related lung cancer diseases among smokers are three times higher than non-smokers of the same population [46]. As reported by Darby et al. [47] cumulative risk of lung cancer when reaching the age of 75 years is estimated to never smokers at 0.4 %, 0.5 % and 0.7 % for the radon concentrations of 0, 100 and 400 Bq / m^3^, respectively. While cumulative risk of lung cancer at the age of 75 years for smokers, reaching 10 %, 12 % and 16 % for radon concentrations of 0, 100 and 400 Bq / m^3^ [47].

According to the United Nations Scientific Committee on the Effects of Atomic Radiation, radon is responsible for about 20 % of all lung cancer diseases in the world. Exposure to radon is the second leading risk factor, causing approximately 21,000 cases of lung cancer per year in the United States [48]. British Radiation Protection Bureau reports that in the UK 1400 people die each year from radon-induced lung cancer [49]. Case–control study carried out in Spain showed that the risk of lung cancer with radon exposed to 147 Bq / m^3^ increase in two times [36]. According to the research of the Canadian scientists, the relative risk of lung cancer in non-smokers living with radon exposed to 200 Bq/m^3^ is doubled and compared with the control group [50]. Peterson and colleagues [51] showed that in Ontario (Canada), 13.6 % of deaths from lung cancer caused by radon action. Based on the assessment the Government of Canada revised the guideline for the exposure to radon in indoor air from 800 Bq / m^3^ to 200 Bq / m^3^ in June 2007 [52]. The population study in Russia has shown that people who were exposed to radon had significantly increased risk of lung cancer [53]. Similar results were also obtained in the study of the risk of radon-induced lung cancer in Chihuahua (Mexico) [54]. In Denmark, from 1993 to 1997 the living conditions of 57.053 people studied, and analysis of the results showed a positive correlation between the incidence of lung cancer and radon indoors [55]. Thus, epidemiological studies conducted in different countries, indicate the existence of an association between a high content of radon and the risk of lung cancer.

However, the problem of the health effect of radon on the Kazakhstan population is poorly understood. Studies elucidating potential health risk among population exposed to radon and genotoxic effect of radon in Kazakhstan are limited or absent in some areas.

### Indoor radon data in the East and North Kazakhstan

The rocks with high radioactivity and tectonic faults with the high emanation can lead to a significant increase of radon concentration indoor and its contribution becomes dominant in the collective dose of the population. According to the experts the contribution of natural sources in the average annual radiation dose of the Kazakhstan population currently stands at 80 %, including 50 % from radon. In some settlements in 70 % of the buildings radon concentration exceeds the maximum permissible concentration (200 Bq / m^3^). There are official data showing that the concentration of radon in the soil in some areas of Kazakhstan reaches 300 000 Bq / m^3^, and the concentration of indoor reaches 6000–12,000 Bq / m^3^, which exceeds the maximum permissible concentration in 60 times [56].

In 2005–2007, a three-year project “The study of the negative impact of natural radioactivity (radon) on public health.” was carried out in Kazakhstan by the Ministry of Environment Protection. This study identified the radon areas in the North, East and in the central part of the country. A high level of radon in water sources and in indoor air was detected in these areas. A high value of equivalent equilibrium volume activity of radon (EEVA) was revealed in East Kazakhstan. Instantaneous value of radon EEVA surveyed localities reaches 8000–9000 Bq / m^3^. Among 222 surveyed facilities (schools, hospitals, houses), the exceeding standards of radon were observed in 71 cases (35 %) [57].

**Fig. 1:**
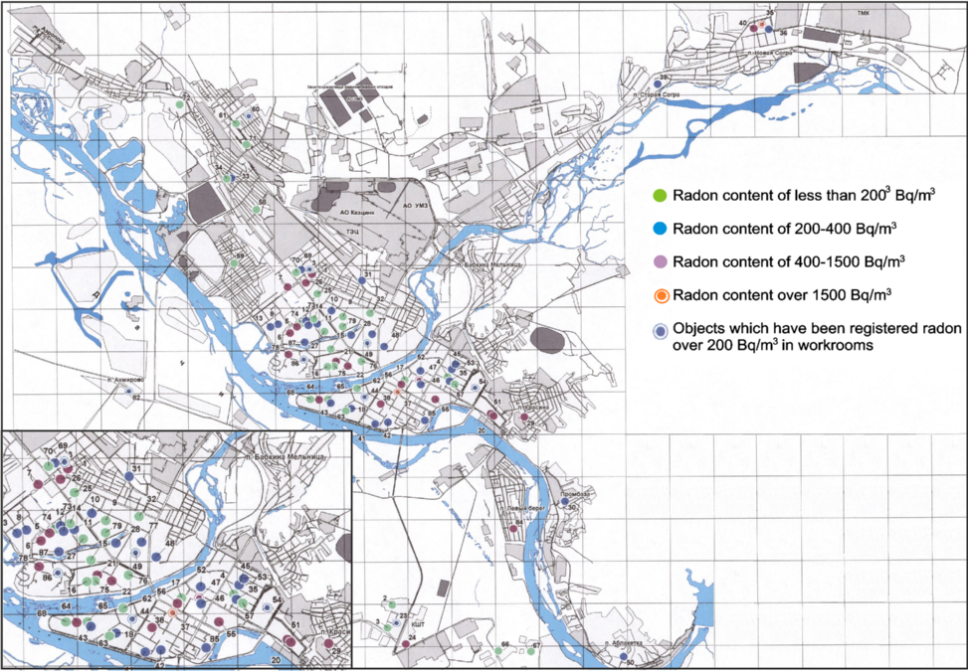
The map of radon in indoor air of Ust-Kamenogorsk (East Kazakhstan) [57]. In the lower left part of the map radon contents (dots) shown in the increased scale

As reported on the official website of the Department of Natural Resources of the East Kazakhstan for the period 1990–1992 in the city of Ust-Kamenogorsk, 137 radioactive anomalies and pollution of various origins with the radiation power in places up to 7000 mR / h, at a rate of 18–20 mR / h were revealed. Measurements of instantaneous values of EEVA conducted in 2005 in the buildings of hospitals, the private homes of the city showed the presence of levels of radon EEVA in the air space in the range of 5 to 2349 Bq / m^3^ (Fig. 1). On 22 objects instant values of equivalent equilibrium volume activity of radon over 200 Bq / m^3^ were recorded in residential and public buildings and on 8 objects EEVA level was more than 340 Bq / m^3^. It is believed that the level of effective dose of the East Kazakhstan population due to radon is 1.5 times more than the average in the world [58].

In accordance with the average value of radon EEVA obtained in 2004–2005, the expected annual individual effective dose of internal exposure of Ust-Kamenogorsk population will be more than 5 mSv / year. According to the sanitary rules and regulations in Kazakhstan, this value is characterized as a high level of public exposure from natural sources of radiation and makes it necessary to conduct some anti-radiation protective measures. During the study in the Altai (East Kazakhstan) region, 15 of the 18 settlements were identified with excess of radon concentration (from 200 to 8000 Bq / m^3^) [59].

Stegnar et al. [7] measured indoor ^222^Rn concentrations in 27 selected houses and public buildings in Ust-Kamenogorsk city. According to their data the values of radon is ranged from 22 to 2100 Bq / m^3^, the average concentration of radon was 230 Bq / m^3^. The corresponding annual effective doses ranged from 0.5 to 20 mSv, the average dose being 5 mSv per year.

The territory of North Kazakhstan is characterized by the areas with the high levels of radiation, arising both from natural radiation sources, as well as by long-term and the large-scale activities of uranium mines and uranium processing companies [60].

In Akmola province located a large region one of the world’s largest North Kazakhstan uranium ore province. It contains more than 30 uranium deposits [61]. More than 50 years in North Kazakhstan being open and underground mining of uranium ore were resulted in the region where has accumulated a significant amount of radioactive waste with the high-risk as a source of radioactive contamination of the environment and harmful to human health. All these factors contribute to the formation of elevated concentrations of radon in the region. The measurement of the radon activity in indoor air was carried out in 2010 on the territory of three districts of Akmola region. As a result of this work there have been revealed 47 settlements, including the new capital of Kazakhstan Astana city and Kokshetau city, 35 settlements (76.1 %) which were characterized by excess of standard values (200 Bq / m^3^) radon activity [56].

**Fig. 2:**
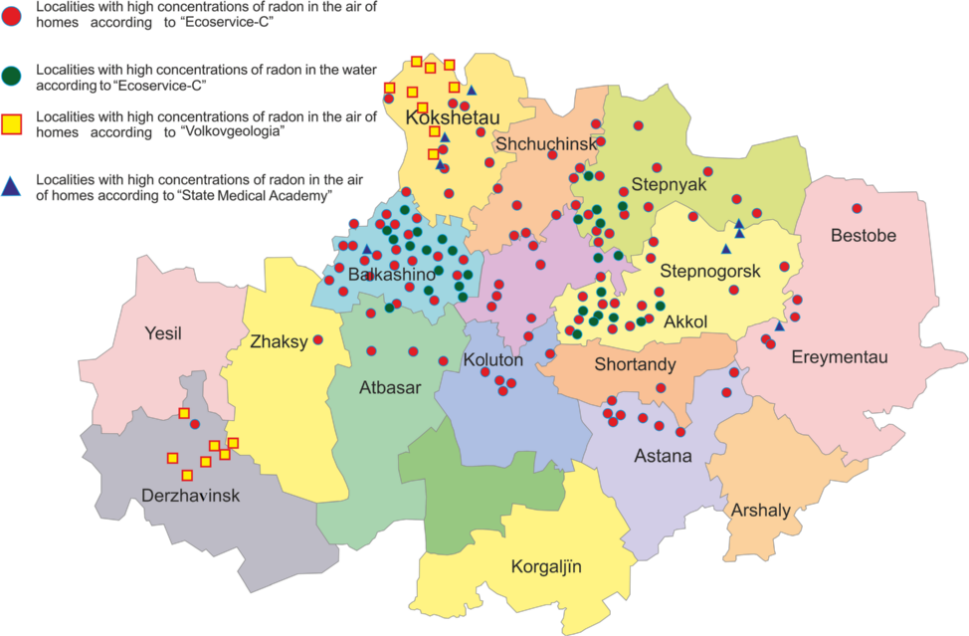
The level of radon in North Kazakhstan [56]. Measurements of radon concentrations conducted by three independent organizations: the State Medical Academy, “Ecoservice С” and “Volkovgeologiya”

In 2010, 814 measurements of radon in indoor air of the residential and public buildings were carried out in Astana in order to implement the preventive health surveillance. Exceeding levels of radon has been established in eight newly built homes (207–1150 Bq / m^3^). It was also revealed the excess of radon activity in the village school Balkashino (1022 Bq / m^3^) and in the preschool of the village “Shantobe” (789 Bq / m^3^). State Medical Academy revealed maximum values ^222^ Rn activity in the settlements: Vasilkivka - 866 Bq / m^3^, Granite - 611 Bq / m^3^, Ondiris - 419 Bq / m^3^. In the village of Aksu, there were 42 surveyed areas in which 22 cases were detected exceeding the permissible radon activity. Activity of radon in homes ranged from 8 to 858 Bq / m^3^, in schools - 153–2162 Bq / m3 and in the basement - 130–5870 Bq / m^3^. In the village Saumalkol the value of radon activity was in the range from 590 to 3977 Bq / m^3^. In some other areas of the North Kazakhstan region (Akkol, Yenbekshilder and Sandyktau) 112 settlements were examined (Fig. 2). More than 60 % of the settlements were identified with exceeding the activity of radon in indoor air and the drinking water sources in 32 % of villages. The most famous case of a high level of radon in mines and in a residential area in Kazakhstan is mine “Akchatau”. It is under a comprehensive survey of radon in mines and homes, as well as the health of miners and residents. As a result, it has been found that the incidences of respiratory diseases, nervous system, cardiovascular system exceed the average for the region is 2.9 times. It is most likely indicative of radon exposure on the health of the village. It should be noted that such studies, unfortunately, are rare in Kazakhstan and in insufficient quantities [56].

### Radon concentrations in drinking water in the East and North Kazakhstan

The active study of the concentrations of the natural radioactive elements in natural waters in Kazakhstan began in the late 40's of the last century due to an increase in geological prospecting for radioactive materials. From 1960 to 1992 the entire territory of Kazakhstan was covered the hydrogeological surveys for water supply of the settlements. To carry out these activities in large volumes, the strictly regulated selections of water samples were conducted for determination of uranium, radium and radon. The amount of available information in the archives and reports of specialized organizations includes information on approximately 30,000 water sources [10]. It has been shown that in the drinking water of some settlements specific activity for ^238^U reached 96 Bq / l, activity for ^226^Ra 45 Bq / kg, and activity for ^222^Rn 5100 Bq / kg respectively [62].

The radiological evaluations of drinking water were carried according the norms established by “Sanitary requirements for radiation safety” representing the main technical document regulating norms of ionizing radiation in Kazakhstan. According to this document, the contents of the natural and artificial radionuclides in drinking water, creating an effective dose of less than 0.1 mSv /year, does not require measurements to reduce the radioactivity of drinking water [63]. Over the past five years in Akmola region (North Kazakhstan) were investigated 1271 drinking water samples, from which 550 samples did not meet the standards for total alpha activity (43 %). On average, the radiation dose of the population received drinking water is 0.21 mSv /year in the region [56].

Thus, the studies of groundwater sourсes in North Kazakhstan have shown the presence of radon in drinking water sources from 8 to 300 Bq / l. The 32 surveyed drinking sources in 15 cases (47 %) had the high levels of radon in drinking water [56].

The studies of groundwater in Ust-Kamenogorsk (East Kazakhstan), conducted in 2005, showed fluctuations in the level of radon (^222^Rn) in the range of 8.0 Bq / l to 81 Bq / l at a rate of radon in drinking water of 60 Bq / l [56].

The concentration of radon in drinking groundwater may be associated with the presence of water in its long-lived daughter product ^210^Pb, which is one of the most radiotoxic beta-active natural radionuclides. Apart from the natural radioactivity of soil, a main source of radon in the environment is a waste of uranium industry.

### Health effect of radon and uranium on the population in Kazakhstan

The analysis of medical statistics of North Kazakhstan showed that the level of overall mortality and cancer among adults and children is one of the highest in the region [64], although it has not revealed a direct relationship between these indicators and the radiation factor as a result of uranium mining and processing enterprises.

The production of uranium involves a large contingent of workers whose work takes place in the specific conditions exposing to radiation and chemical factors present in the ore mined. Klodzinskiy A. and colleagues [65] have shown a deterioration of health at uranium miners in North Kazakhstan. The results of epidemiological and medical studies find out the high frequency of respiratory diseases (61 %) in the cohort of uranium miners of North Kazakhstan. The main form of pathological changes was degenerative disorders in the form of atrophic rhinopharyngitis [65].

According to Djumasheva R. and Kazymbet P. [66] the most common somatic diseases among uranium workers of Stepnogorsk city were hypertension, chronic obstructive pulmonary disease and chronic gastritis. Moreover dysfunction of all parts of the immune system was observed in the studied groups of uranium workers [67]. Comprehensive clinical assessment of health status of children and adults living near Stepnogorsk uranium processing enterprises showed a high risk of developing chronic diseases of internal organs due to long-term toxic effects of radiation. The study of reproductive health of women living in areas adjacent to the uranium mines showed a low percentage of healthy women (13 %) [68]. Among children of Stepnogorsk city the urinary tract infections and chronic bronchitis amounted to a high percentage of the total somatic pathology’s number [69].

In our previous study [70, 71] uranium mine-workers in the Stepnogorsk mining-milling complex in North Kazakhstan were investigated for the expression of chromosome aberrations and for genetic factors that can modify the exposure related expression of chromosome damage. The study has demonstrated an increased frequency of chromosomal aberrations in uranium miners compared to the matched control subjects, which were not exposed to uranium compounds or to any other known chemical or physical mutagens. The data on aberration type’s frequency showed a predominance of aberrations of chromosome type in the exposed group, mainly including paired acentric fragments, dicentric chromosomes and centric rings. The study showed the dependence of the chromosomal aberration frequency on length of service in uranium mines.

The uranium workers were classified into 3 groups according to the duration of exposure: group I – 1–10 years, II – 11–20 years and III – 21–25 years. The received data show that all three groups of workers had higher frequencies of chromosomal aberrations than the control group. In the first group of workers the frequency of dicentrics was higher (1.95 ± 0.15) than that for the matched control group (0.50 ± 0.09) (0.05 < *p* < 0.01). In the second group of uranium workers the frequency of dicentrics and the total chromosomal aberrations were significantly higher (2.33 ± 0.17) than in control group (0.55 ± 0.08). In the third group of workers the frequency of chromosomal aberrations was also significantly higher (2.68 ± 0.26) than in control group (0.36 ± 0.10). The frequency of chromosomal aberrations observed in this exposed group III was also higher compared with the first and the second exposed groups (0.05 < *p* < 0.01) [72].

Furthermore it was observed a significant increase in the frequency of chromosomal aberrations in the workers who were also heavy smokers compared with those who were moderate smokers or non-smokers. Uranium exposed workers who had inherited the null GSTM1 and/or GSTT1 genotypes had a significant increase in the frequency of chromosome aberrations compared with those who had intact GSTM1 and GSTT1 genes for different group of workers. We have shown that the uranium mining conditions in North Kazakhstan can cause a long-term health risk among the workers.

In uranium miners were observed the various forms of malignant tumors of the lung, liver and stomach [73].

**Fig. 3:**
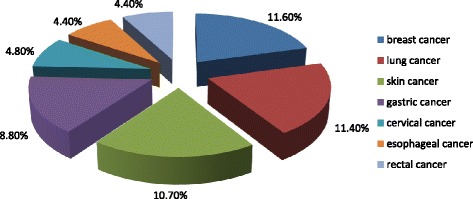
Structure of cancer in Kazakhstan. (Data of Kazakh Research Institute of Oncology and Radiology, 2011) http://svant.yvision.kz/post/361082?utm_source=digest20130907&utm_medium=email&utm_campaign=digest

Among human cancers in Kazakhstan, the lung cancer is in the first place (Fig. 3), which represents 11.4 % of the number of cancer patients, while in the structure of morbidity among men leading positions are occupied by the tumor of the trachea, bronchus, and lung (20.4 %) (unpublished observations). During the period from 2002 to 2011 were registered 37,241 lung cancer patients in the Republic of Kazakhstan. Among lung cancer patients was 30 554 male (82.0 %) and 6687 female (18.0 %) and the ratio was 4.6: 1.0 [74]. In North Kazakhstan there is a highest frequency of cancer in comparison with the other regions of the country [75].

Over the past 30 years in East Kazakhstan there was a sharp increase in cancer rates. This growth is particularly noticeable for the Ust-Kamenogorsk city. The increase in total cancer morbidity indicator is mainly due to an increase in the incidence of lung cancer, breast cancer and skin cancer [76].

As mentioned above, the villages where there is a high radon activity, marked by the territory of Kazakhstan, but are characterized by the highest levels of radon East and North Kazakhstan (67 % of settlements in excess). Interestingly, it is in the Eastern and Northern part of the country there is increased (up to 1.5 times or more) the cancer rates, which makes it possible to assume a correlation between radon levels and cancer incidence [76].

Unfortunately, it has been not enough research of the influence of radon on the risk of cancer for the population in Kazakhstan. In the study of Priest et al., no association between radon and childhood leukemia was found in south Kazakhstan and northern Kyrgyzstan, but the radon concentration was too low (in Kazakhstan was 123 Bq /m^3^ and in Kyrgyzstan was 177 Bq /m^3^) [77].

The strange sleeping sickness in the Kalachi village (North Kazakhstan) was first registered in March 2013 [78]. For two years, people have been experiencing these sudden urges to fall asleep and some people remained sleeping for as long as two days. In January 2015 above one hundred people, including children of Kalachi village, diagnosed with “encephalopathy of unknown etiology” were taken to hospital. Kalachi is located in the vicinity of Krasnogorsk city, where uranium ore mined during the Soviet period. One of the most popular versions of this strange sleeping symptom is the uranium poisoning and the influence of radiation. The experts produced 200 measurements of gamma background outdoor and 100 indoor. The value of gamma background was 0, 08–0, 14 μSv / h (at a rate of 0.3 μSv / h). Measurements carried out in homes which showed radon levels from 226 to 567 Bq / m at a rate of 200 Bq / m^3^. However, the cause of the disease has not been established until now [78].

Influence of environmental factors including radon on cancer development among population of North and East Kazakhstan regions were investigated by group of researchers from North Kazakhstan State University. They have found a positive association between residential radon concentration in these two regions of Kazakhstan and different cancer diseases, predominating lung cancer [76].

The problem of radon areas in Kazakhstan is currently defined as one of the most important environmental health problems of the population.

## Summary and conclusion

Many published data have demonstrated the potential of radon exposure to induce biological damage. All these studies suggest that exposure to radon and its progeny can represent a significant public health risk. Radon exposure is considered as the second cause of lung cancer and it is a first in never smokers. Many countries have depicted residential radon exposure maps in order to characterize those areas with the highest indoor radon concentrations.

There are areas in Kazakhstan with a number of factors leading to natural and man-made elevated radioactivity, including numerous sites abnormally elevated natural radioactivity, uranium deposits, as well as long-term activity of uranium mines and mining enterprises other minerals associated with uranium mineralization. Therefore, it becomes clear that exposed human populations should be monitored for risk assessment and disease prevention. Since this topic is very relevant for the Republic of Kazakhstan, it is necessary to conduct the full-scale studies on the risk of radon-induced lung cancer in population living in areas with high levels of radon. These studies conducted in compliance with all requirements of genotoxic studies will provide the extensive and reliable data for a detailed radon zoning of Kazakhstan. It will also provide an opportunity to plan the construction of residential and industrial buildings with the low radon risk exposure. This in turn will help to develop measures to reduce the risk of radon exposures on the health of people living in areas with elevated radon concentrations in the air.
